# COVID-19: sleep research perspectives

**DOI:** 10.5935/1984-0063.20200033

**Published:** 2020

**Authors:** Arezu Najaﬁ, Khosro Sadeghniiat-Haghighi, Zahra Banafsheh Alemohammad, Samaneh Akbarpour

**Affiliations:** Occupational Sleep Research Center, Baharloo Hospital, Tehran University of Medical Sciences, Tehran, Iran.

**Keywords:** Sleep, Coronavirus Infections, Research

## Abstract

Coronavirus disease of 2019 (COVID-19) pandemic originated from Wuhan in December 2019 and has been spread in whole China and the world. Worldwide outbreaks of COVID-19 triggered a large number of morbidities and mortalities beside its economic and social burdens, which are discussed a lot, in scientiﬁc literature and different types of media. As a sleep medicine specialist, we may wonder how we can be involved in this ﬁeld. In this short theoretical essay, we will discuss about the known facts as well as the hypotheses, which associate the sleep medicine to COVID-19. The discussed points may provide a source of research ideas throughout the world for better understanding of novel coronavirus-19 that has devastating effects on humanity.

Coronavirus disease of 2019 (COVID-19) pandemic originated from Wuhan in December 2019 and has been spread in whole China and the world. Worldwide outbreaks of COVID-19 triggered a large number of morbidities and mortalities beside its economic and social burdens, which are discussed a lot, in scientific literature and different types of media. As a sleep medicine specialist, we may wonder how we can be involved in this field. In this short theoretical essay, we will discuss about the known facts as well as the hypotheses, which associate the sleep medicine to COVID-19. The discussed points may provide a source of research ideas throughout the world for better understanding of novel coronavirus-19 that has devastating effects on humanity.

The first scientific point, which comes to the mind regarding association of COVID-19 and sleep medicine, is the role of healthy sleep in boosting immune system. There is a proven fact that indicates sufficient and healthy sleep is required for appropriate response of immune system against pathogens^1-5^.

The other potential association is the role of known sleep disorders as a risk factor for COVID-19. It is supposed that shift workers and patients with insomnia have increased risk for this disease; although more investigation is needed to support this hypothesis. We have a group of patients with various sleep disorders in our clinics; screening of the COVID-19 symptoms would be elucidative in this regard. The most common patients attending our clinics as a referral center are patients with obstructive sleep apnea (OSA). The question that arises here is if OSA with recurrent episodes of intermittent hypoxemia and sleep fragmentation during the night would be a risk factor for COVID-19. Up to now, we know that middle-aged men and obese people are at increased risk for developing COVID-19^6,7^, on the other side, OSA is more prevalent in this population^8^. Can OSA be a risk factor and is there any association between severity of overnight oxygen desaturations, respiratory disturbance index (RDI), and COVID-19? This question needs attention of sleep specialists and exploratory researches.

Additionally, some studies considered blood group type A as a risk factor for coronavirus-19^9^. The evidence supports the hypothesis of more prevalence of mood disorders including anxiety in people with blood type A^10,11^. Anxiety disorder is one of the main consequences of insomnia and other related sleep disorders. The hypothesis that the patients with blood group A may suffer from more sleep disorders including insomnia is another interesting area of research regarding the association between COVID-19 and sleep medicine. Moreover, adverse effects of lockdown and isolation on sleep pattern is an interesting field of research during pandemic of COVID-19. For instance, COVID-19 related mental health problems, economic impact of lockdown, and irregular sleep-wake cycle influenced by changes in environmental cues of circadian system, may result in disturbed sleep and insomnia.

In our opinion, the most intriguing connection between COVID-19 and sleep medicine is the hypothesis that virus attacks central nervous system (CNS) directly by adhesion to ACE2 receptors in different parts of CNS^12,13^. Neurologic symptoms including sleepiness have been reported in patients with COVID-19^14^. Patients also report sleepiness and even sleep attacks during the course of the disease. The presence of ACE-2 receptors in sleep-wake centers of CNS including anterior hypothalamus, ventral lateral preoptic (VLPO) area of the hypothalamus, and suprachiasmatic nucleus as the center for circadian system and thermoregulation makes the association of sleep medicine and COVID-19 more interesting. Hypoxemia as a result of COVID-19 and sleepiness as a consequence may have an association together. Therefore, sleepiness can be used as a follow up measure of COVID-19 severity. This area is also an intriguing field of sleep medicine research.

In conclusion, looking at the COVID-19 and sleep medicine, we identified some potential and interesting research agenda, which sleep specialists, can use along with the battle of the world in the fight against COVID-19.
